# Acute superior mesenteric venous thrombosis with advanced gastric cancer: a case report

**DOI:** 10.1186/1757-1626-3-76

**Published:** 2010-03-09

**Authors:** Fuminori Goda, Hiroyuki Okuyama, Ayumu Yamagami, Hiromi Nakata, Michio Inukai, Eiji Ohashi, Takeaki Kohno, Takashi Himoto, Hisashi Masugata, Shoichi Senda

**Affiliations:** 1 Cancer Center, Kagawa Medical University Hospital, 1750-1 Ikenobe, Miki-cho, Kita-gun, Kagawa, 761-0793, Japan; 2 Department of Integrated Medicine, Kagawa Medical University Hospital, 1750-1 Ikenobe, Miki-cho, Kita-gun, Kagawa, 761-0793, Japan

## Abstract

Although the advanced stages of neoplasms have a risk of superior mesenteric venous thrombosis (MVT), an initial clinical diagnosis of MVT is sometimes difficult and it can be treated as a cancer-related pain using NSAIDs and/or opioids.

We herein present a case of palliative stage of cancer with acute MVT, which was successfully treated with immediate anticoagulant therapy. We believe this case provides an important clinical lesson, which is that we should remember that MVT is one of the potential causes of abdominal pain with cancer patients and the thrombosis can be easily identified by US and CT.

## Introduction

Although the advanced stage of the neoplasm has a risk of superior mesenteric venous thrombosis (MVT) [1], an initial clinical diagnosis of MVT is sometimes difficult and it can be treated as a cancer-related pain. We herein present a case of palliative stage of cancer with acute MVT, which was successfully treated with immediate anticoagulant therapy. His clinical course suggests we should remember that MVT is one of the potential causes of abdominal pain with cancer patients and the thrombosis can be easily identified by US and CT.

## Case presentation

A 69-year-old male was admitted because of a continually worsening abdominal cramping pain, which had started two days before his admission. The pain was radiating to his back and it was associated with nausea and appetite loss. The patient had previously undergone a total gastrectomy for advanced gastric cancer two years earlier.

**Figure 1:**
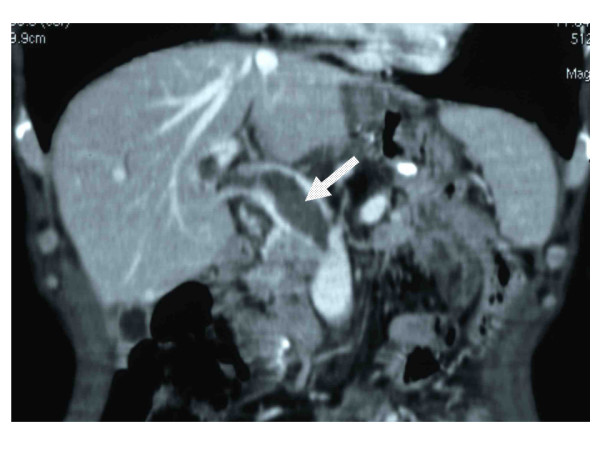
**Contrast-enhanced abdominal CT**. The thrombosis located in the superior mesenteric vein with portal vein (arrow) without cavernous formation.

**Figure 2:**
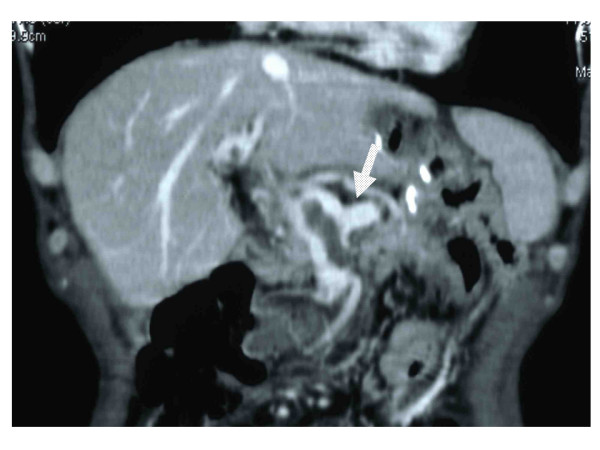
**Contrast-enhanced abdominal CT**. No thrombosis was observed in the splenic vein (arrow).

Thereafter, peritoneal relapse occurred and chemotherapy with paclitaxel and S-1 was administered by the outpatient department for six months with uneventfully. At admission, he was fully alert and his vital signs were normal, although he had mild grade fever (37.5°C). On examination, his abdomen was soft and flat with abdominal tenderness at the epigastrium, with diminished bowel sounds. Biochemical results and coagulation studies were within the normal limits. However, his symptoms were getting worse and opioid titillation was thus initiated. He underwent abdominal ultrasonography (US), which showed evidence of thrombosis or tumor thrombosis in the portal vein. A contrast-enhanced CT scan showed evidence of superior mesenteric venous thrombosis associated with thrombosis of the portal vein without cavernous formation(Figure 1). There was no thrombosis in the splenic vein (Figure 2). The dehydration was corrected by the fluid therapy. Anticoagulant therapy was started, because the thrombosis had formed recently and the risk of bleeding was low. The patient was given low-molecular-weight heparin (Dalteparin sodium, Fragmin) at a dosage of 10,000 U per day. Three days after the administration of heparin, he had a complete recovery from abdominal pain without opioid use and was thus able to start eating. He was discharged after 14 days of admission with a shift of the anticoagulant therapy to warfarin with a targeted international normalized ratio (PT-INR) of 1.5-2.0. Three months later, he was doing well and the thrombosis of the superior mesenteric vein and portal vein had completely disappeared on a follow-up CT. Six months later, he died from gastric cancer and the autopsy showed no thrombosis in the superior mesenteric vein and portal vein.

## Discussion

MVT has been assumed to be a very rare disease. The incidence of MVT was estimated at 1.8-2·7 per 100 000 person-years[2,3]. This accounts for 1.5-6.2% of patients of acute mesenteric ischemia in various studies[4]. The neoplasm is among the more common causes of mesenteric venous thrombosis. As illustrated in Table 1, 83 (21%) of 395 cases of MVT associated with neoplasm [2-7]. The advanced stage of the neoplasm has a risk of developing MVT such as, hyper-coagulability, a low flow state in the superior mesenteric vein as a result of dehydration and chemotherapy [5,8,9].

**Table 1:** Incidence of Cancer relative MVT

Authors	term	cases	cancer related cases
Acosta, S(2005) [2]	1970-1982	23	8 (35%)

Acosta, S(2008) [3]	2000-2006	51	12 (24%)

Kumar, S(2003) [4]	1979-1998	69	9 (13%)

Janssen, HL(2001)[5]	1984-1997	172	41(24%)

Sogaard K(2001)[6]	1992-2005	67	9 (13%)

Morasch, MD(2001)[7]	1985-1999	13	4 (31%)

total		395	83 (21%)

The clinical diagnosis of MVT associated with a neoplasm is sometimes difficult. The symptoms and signs of MVT are non-specific, such as abdominal pain with nausea, vomiting, diarrhea/constipation and fever [4], and the initial physical findings may often appear to be entirely normal. These conditions are often assumed to be cancer related symptoms, which can be treated with NSAIDs and/or opioids. In the present case, the initial diagnosis was cancer-related pain during chemotherapy for gastric cancer with peritonitis carcinomatosa.

The use of advanced imaging techniques provided useful and helpful information. US is a powerful modality for the screening of a suspected thrombosis. Contrast-enhanced CT, which facilitates the early and accurate detection of thrombosis, including fresh thrombosis with a high sensitivity [4], therefore indicated the precise location of the thrombosis and the duration of the thrombosis in this case.

The best strategy for MVT is its early diagnosis and immediate treatment [1]. Anticoagulant therapy, which generally allows for the re-canalization of the thrombosed vein in recently formed thromboses, is recommended in patients with acute MVT to improve the survival without increasing the risk of bleeding [4,10], however, reliable data for the palliative stage of cancer patients with MVT is still scarce.

## Conclusion

The palliative stage of a cancer patient with MVT was successfully treated. It is important to remember that MVT is one of the causes of abdominal pain in cancer patients and such thrombosis can be easily identified by both US and CT. Even in palliative stage cancer patients, the immediate use of anticoagulants can improve both the QOL and the prognosis of the patients.

## Consent

Written informed consent was obtained from the patient for publication of this case report and accompanying images. A copy of the written consent is available for review by the Editor-in-Chief of this journal.

## Competing interests

The authors declare that they have no competing interests.

## Authors' contributions

FG managed the case with full responsibility, and wrote manuscript. HO, AY, TK, TH, HM treated the patient and interpreted the patient data. HN, MI, EO contributed to the writing and editing of the manuscript. SS was the general consultant and advised in the management of the case and writing of the manuscript. All the authors read and approved the final manuscript.
